# The Development of Musical Skills of Underprivileged Children Over the Course of 1 Year: A Study in the Context of an El Sistema-Inspired Program

**DOI:** 10.3389/fpsyg.2016.00062

**Published:** 2016-02-02

**Authors:** Beatriz S. Ilari, Patrick Keller, Hanna Damasio, Assal Habibi

**Affiliations:** ^1^Music Teaching and Learning, Thornton School of Music, University of Southern CaliforniaLos Angeles, CA, USA; ^2^School of Medicine, Vanderbilt UniversityNashville, TN, USA; ^3^Brain and Creativity Institute, Dornsife College of Letters, Arts and Sciences, University of Southern CaliforniaLos Angeles, CA, USA

**Keywords:** musical development, middle childhood, underrepresented populations, longitudinal study, El Sistema-inspired programs

## Abstract

Developmental research in music has typically centered on the study of single musical skills (e.g., singing, listening) and has been conducted with middle class children who learn music in schools and conservatories. Information on the musical development of children from different social strata, who are enrolled in community-based music programs, remains elusive. This study examined the development of musical skills in underprivileged children who were attending an El Sistema-inspired program in Los Angeles. We investigated how children, predominantly of Latino ethnicity, developed musically with respect to the following musical skills – pitch and rhythmic discrimination, pitch matching, singing a song from memory, and rhythmic entrainment – over the course of 1 year. Results suggested that participation in an El Sistema-inspired program affects children’s musical development in distinct ways; with pitch perception and production skills developing faster than rhythmic skills. Furthermore, children from the same ethnic and social background, who did not participate in the El Sistema-inspired music program, showed a decline in singing and pitch discrimination skills over the course of 1 year. Taken together, these results are consistent with the idea of musical development as a complex, spiraling and recursive process that is influenced by several factors including type of musical training. Implications for future research are outlined.

## Introduction

Musical development is a complex and dynamic, non-linear process that is spiraled and recurring (Bamberger, 2005). It is influenced by a wide range of interrelated factors, including maturation of the brain and body, cultural practices and values, and degree and quality of engagement with music through formal and informal learning experiences (see Lamont, 1998; Hargreaves et al., 2003; McPherson, 2009, 2016; Mapana, 2011; Putkinen et al., 2014; Ilari and Habibi, 2015). Although there is some agreement that musical development occurs across the lifespan, most research conducted to date centers on changes that occur between childhood and adulthood (Gembris, 2006). Childhood is a time of rapid cognitive, emotional, social, neural and motor changes. Therefore, studying musical development during this period of life allows researchers to examine whether there are normative changes to musical abilities and skills across time (see Corrigall and Schellenberg, 2016), and to consider how such knowledge can be applied in educational and therapeutic settings (Hargreaves et al., 2003).

In recent years, scholars have turned their attention to the social and cultural contexts that may either enable or hinder children’s musical development. That is, the tendency that was prevalent in the past of conceptualizing children’s musical development based mainly on the observation of age and time related changes in specific musical skills, and without much consideration to their enabling social and cultural contexts (e.g., Shuter-Dyson, 1968; Swanwick and Tillman, 1986; Zimmerman, 1986; Serafine, 1988), is being gradually replaced by a more contextualized and integrated view of musical development. These new advances in the field are taking place in response to criticisms that were drawn at musical development research. One such criticism concerned study samples, as much developmental research in music has been conducted predominantly with populations from what Heinrich et al. (2010) called WEIRD societies (i.e., white, English speaking, intelligent, and from rich and democratic countries). Little research exists to date concerning the development of musical skills in children from diverse social, ethnic and cultural groups. This is also true for the lack of such research in rich, industrialized and democratic societies where musical development research has a strong tradition (e.g., United States, UK). Therefore, what is known about the development of musical skills in early and middle childhood is heavily based on research conducted with children from middle class families in Western societies, many of whom with access to music in varied ways, including through specialized learning programs in schools and conservatories.

Another related point of criticism of developmental research in music has been the lack of investigations on children’s musical skills in community-based programs. While some recent studies are beginning to fill in this gap (e.g., Rauscher and Hinton, 2011; Kraus et al., 2014; Welch et al., 2014a,b, 2015; Osborne et al., 2015; Slater et al., 2015; Tierney et al., 2015), it is interesting that most have focused primarily on non-musical outcomes (e.g., mathematical abilities, reading skills, self-regulation, resilience, and speech perception). Although there are some exceptions (e.g., Welch et al., 2014a,b), children’s musical skills are typically not reported in most studies. This is unfortunate, given the scarcity of research on the development of musical skills of children from lower SES, including many who take part in El Sistema and El Sistema-inspired programs, which are becoming increasingly popular across the world.

### El Sistema and El Sistema-Inspired Music Learning Programs

Acknowledged worldwide as the “most significant example of collective music education” (Majno, 2012, p. 56), El Sistema is claimed by many to be an important mechanism of social change through music (Booth, 2012; Majno, 2012). Since its inception in 1975, 1000s of children, most from underserved communities, have gone through this publicly funded program in Venezuela. Through intensive, collective music learning experiences, El Sistema has been said to promote inclusion and combat poverty by empowering at-risk children and youth, and providing them with high quality music learning experiences (see Sánchez, 2007; Uy, 2012). El Sistema *núcleos* (schools) are described as inclusive—as they are open to all—and operate somewhat independently from one another. Participation in ensembles, choirs and bands is mandatory and viewed as a way for students to develop musical abilities, negotiate identities and redefine communities, all within a safe space (Pettigrew, 1998; Sánchez, 2007; Uy, 2012).

The promise of El Sistema as a mechanism for social transformation and potential for reaching large groups of students at once, allied with the major cuts to public school music education in many nations, has propelled the creation of El Sistema-inspired music education programs throughout the world (Majno, 2012; Uy, 2012). Osborne et al. (2015) argue that the El Sistema approach is highly adaptable, which helps to explain its appeal. In the United States over 60 programs (Hulting-Cohen, 2012) were identified at the time of writing. These programs are offered by non-profit organizations alone or held in partnerships with public and charter schools, community organizations and universities. Like in Venezuela, most El Sistema-inspired programs in the U.S. serve children and youth from lower SES (Hulting-Cohen, 2012). Course offerings and practices vary considerably from one program to next (e.g., Osborne et al., 2015) and this is due to the individualized needs and cultural contexts of the communities where they stand. Yet, a point of convergence between these programs lies in the ethos of the El Sistema approach or the idea of music as a means to develop musical and extra-musical abilities in children from disadvantaged backgrounds (Uy, 2012).

El Sistema and, likewise, El Sistema-inspired programs, are not free of challenges (Majno, 2012) and criticisms (e.g., Baker, 2014). Ideological beliefs aside, an important criticism that has been aimed at them is the lack of assessment of their students in musical and non-musical domains (see Majno, 2012; Uy, 2012; Baker, 2014). That is, many claims are often made in regards to children’s gains in terms of musical, social, and cognitive development as a result of their participation in these programs (for discussions see Booth, 2009, 2012; Majno, 2012; Baker, 2014), but little empirical data exists to date to substantiate or refute them. This was one important motivation to carry out the present study. On a more specific note, our aim was to gage the development of musical skills in children attending an El Sistema-inspired program in Los Angeles, CA, USA. Given that the curricula of El Sistema-inspired programs are usually customized to the needs of individual communities, a dilemma that we faced lay in determining the musical skills to be assessed. From the start, we were determined to assess a variety of musical skills, as research in the field has typically focused on single skills. Additionally, it was important for the selected skills to be aligned to the curriculum of the program that we were studying, and to be examined in light of earlier musical development studies.

Among the gamut of musical skills that are known to develop in childhood (Hallam, 2016), we decided to focus on pitch discrimination, rhythmic discrimination, rhythmic synchronization, and singing. These four skills are known to develop considerably and to plateau in middle childhood (Zimmerman, 1986; Gooding and Standley, 2011), a period in time when children become increasingly independent and musically sophisticated (Corrigall and Schellenberg, 2016). Pitch and rhythmic discrimination are arguably central to music learning, particularly where instrumental music education through western “art” music is concerned. Rhythmic synchronization, in turn, is essential for the experience of music (Corrigall and Schellenberg, 2016), including for playing in ensembles. In spite of its importance for musical experiences, the development of rhythmic synchronization has received little attention from the scholarly community, and this why it was examined in our study. Singing tasks were also added because singing is a behavior that most children engage in, to some extent, in everyday life. Unlike performance on a musical instrument, singing skills could be assessed in all child participants. Our review of previous research concerning these four skills centers on middle childhood (see Eccles, 1999), more specifically between 6 and 10 years, which reflects the age of our study participants, as we will discuss ahead.

### The Development of Four Specific Musical Skills in Middle Childhood

A central feature of music perception and cognition (Krumhansl, 2000), pitch is a salient attribute that humans perceive from very early on (Oxenham, 2012). The perception of musical pitch involves a wide range of components including frequency discrimination, pitch change detection, and sensitivity to pitch direction, to name a few. According to Fancourt et al. (2013), the latter two are particularly important for music as they are implicated in the perception of melodies, which, in turn, forms the basis of much Western tonal music. Interestingly, both frequency discrimination and pitch change detection reach adult levels only when children are about 6–7 years of age (See Gooding and Standley, 2011). Sensitivity to pitch direction, however, does not become adult-like until children are around 10 or 11 years of age (Trainor and Corrigall, 2010; Fancourt et al., 2013). Similarly, while some building blocks of harmonic perception are already seen in infancy and during the preschool years (Costa-Giomi, 2003; Corrigall and Trainor, 2010), this particular skill takes time to develop, becoming adult-like solely when children are between ages 10–12 (Trainor and Corrigall, 2010).

The development of the perception and production of temporal events in childhood also develops considerably during middle childhood. These two different yet related skills are often placed under the same heading due to the embodiment of rhythmic perception (Trainor and Corrigall, 2010). Unsurprisingly, children’s responses in tasks concerning the perception of temporal events in music are often examined by means of observed body movements and gestures such as tapping, clapping, marching or synchronizing to the beat (e.g., Upitis, 1987; Gerard and Drake, 1990; Drake, 1993; Drake et al., 2000; Reifinger, 2006; Kirschner and Tomasello, 2009).

There are two basic perceptual organizational processes related to the encoding, retrieval and production of rhythmic patterns, namely, grouping and derivation of metrical structures (see Trainor and Corrigall, 2010). Grouping of metrical structures refers to what musicians typically call rhythmic patterns or phrases, with their own beginnings and endings. Derivation of metrical structures refers to the ability to perceive and extract underlying beat hierarchies in a rhythmic pattern, or what musicians call musical meter. The derivation of metrical structures and, moreover, the ability to synchronize one’s body gesture/voice to the musical beat (i.e., rhythmic synchronization) is known to be a complex skill that makes demands on auditory, perceptual, analytical and motor functions (see Phillips-Silver et al., 2010). Both the perception and production of temporal events develop considerably during childhood. Age (Drake et al., 2000), musical training (Drake et al., 2000) and culture (Kirschner and Ilari, 2014) are known to influence children’s discrimination of accents in rhythmic patterns (Gerard and Drake, 1990), reproduction of rhythms (Gerard and Drake, 1990), ability to keep a steady beat (Upitis, 1987), and rhythmic synchronization (Kirschner and Ilari, 2014). Importantly, rhythmic synchronization is a socially learned behavior that is influenced by culture, stimulus properties, individual dispositions, and children’s motor skills, including degree of control of their bodies.

Singing, in turn, is a highly complex human behavior (Welch, 1994, 2016; Stadler-Elmer, 2011) that is considered both universal (see Nettl, 1992) and central to the development of musicianship (Demorest and Pfordresher, 2015). The act of singing relies on a series of transformations between the motor plan (e.g., muscle movements like respiration, phonation, and articulation), low-level perception (e.g., perception of scales, notes, timbres), and categorical representations (e.g., contribution of musical schemata to low-level perception), with the latter being stored in long-term memory (Pfordresher et al., 2015).

While singing, in the not so distant past, was often treated as an ability of a few “talented” individuals (see Mang, 2006), in recent years, there has been a consensus that singing is actually a developmental skill (Stadler-Elmer, 2011; Welch, 2016). Over the course of child development, sung pitch intervals widen and sung melodies expand in terms of rhythmic and melodic organization (see Winner, 2007). These behaviors have been observed in one of the major milestones of singing development in childhood: the genesis of invented songs that is common to the preschool years (see Winner, 2007; Gooding and Standley, 2011). In terms of singing conventional songs found in the child’s cultural environment, key stability in singing does not emerge until the onset of middle childhood (see Gooding and Standley, 2011). That is, children’s singing of familiar and improvised songs becomes more stable and discrete when they are 6 or 7 years old (Hargreaves and Zimmerman, 1992). Analogously, the capacity to maintain tonal stability while singing usually occurs when children are around ages 7 or 8 (Hargreaves and Zimmerman, 1992).

Developmental studies on singing further suggest that whereas age may play a role in singing accuracy (see Welch, 2016), experience in singing often overrides it (Demorest and Pfordresher, 2015). That is, while singing ranges expand and pitch matching abilities may become more robust over the course of development, the act of singing is dependent on the offer of ample opportunities to engage in and practice these skills (Campbell and Scott-Kassner, 2013). Other factors that are known to affect the developmental course of singing skills are children’s use of vocal registers, gender, language, singing alone or in groups, and type of music instruction (Mang, 2006; Rutkowski, 2015). The nature of singing tasks is yet another important issue to consider. As an example, Mang (2006) argued that pitch matching (imitation) and singing a song from memory, which are common tasks used in developmental singing research, make different demands on the human brain and body, which explains why they are often not correlated. Additionally, the development of singing is a cultural matter (see Ilari and Habibi, 2015). As Welch (1994, p. 5) suggested, “musical judgments about singing are more about ‘goodness of fit’ in relation to the dominant cultures and are normally culture-specific.”

Taken together, the abovementioned studies reinforce the idea of middle childhood as an ideal time for developing specific music skills such as singing, pitch and rhythmic discrimination and rhythmic entrainment. Additionally, an assumption that derives from these and other developmental studies is that engagement in formal music learning programs further accelerates the development of musical skills in children (e.g., Fujioka et al., 2006; Trainor and Corrigall, 2010). Although this assumption is commonsense, it is also vital to acknowledge that music learning programs are qualitatively different in their philosophies, teaching approaches, demands placed on students, and choice of repertoire. Combinations of these factors with student characteristics and dispositions are likely to influence the development of musical skills in distinct ways.

## Purpose of the Study

The purpose of the current study was to examine the development of musical skills in underprivileged children, who were taking part in an El Sistema-inspired program in Los Angeles, California. We investigated how children, predominantly (but not exclusively) of Latino ethnicity, developed musically with respect to the following musical skills—pitch and rhythmic discrimination, pitch matching, singing a song from memory, and rhythmic entrainment—over the course of 1 year, and compared their progress with that of children from the same underprivileged community who did not participate in any structured musical program. The results reported here are parts of a larger longitudinal study of child development related to music learning, performed at the Brain and Creativity Institute with the collaboration of the Los Angeles Philharmonic, Youth Orchestra of Los Angeles, the organization Heart of Los Angeles, and two elementary schools from the same geographical area (see Habibi et al., 2014).

## Materials and Methods

### Participants

Fifty 6- to 7-year-olds were recruited from two local elementary schools and a community-based music program in Los Angeles, CA, USA and formed two groups: experimental and control. The experimental group consisted of 23 children (nine girls, mean age at baseline = 79.3 months, *SD* = 6.6), who were learning music within the Youth Orchestra of Los Angeles at Heart of Los Angeles, also known as YOLA at HOLA^1^ (see description ahead). Twenty-seven children (10 girls, mean age at baseline assessment = 84 months, *SD* = 5.4) formed the control group. Children in the control group were recruited from two local elementary schools from the same geographical area, provided they were not involved in any systematic and intense after-school program. None of these schools provided a comprehensive music education program for their students.

Children in both cohorts were primarily of Latino^2^ ethnicity and came from equally under-served communities. Child participants and their families resided in some of the most densely populated areas of Los Angeles, which are also characterized by social isolation and lack of opportunities for youth, high levels of poverty, violence and gang activity, and public neglect in general. All children were raised in bilingual households, but attended schools in the dominant language (i.e., English).

### Music Learning at YOLA at HOLA: Access and Curriculum

Child participants in the experimental group took part in the El Sistema-inspired program called YOLA at HOLA, which offers free music tuition, 5 days a week, to children from underserved communities of Los Angeles. Aligned with the central tenets of El Sistema that were discussed earlier, the program emphasizes ensemble practice and group performances. To join the program, children were selected by lottery, up to a maximum of 20 per year, from an extensive list of interested families.

The musical curriculum for first year students consisted of 7 weekly hours of music learning, divided into the following activities: violin (3 h), choir (2 h), Orff (1 h), musicianship—ear training and theory skills (1 h)—, and of 4 h of homework tutoring. Children attended the program 5 days a week (Monday through Thursday, and Saturdays) on a regular basis, and also took part in sporadic, extra-curricular activities including varied concerts and performances and master-classes. The curriculum was designed to gradually socialize children into the program and into ensemble performance. As an example, children constructed cardboard instruments, as an initial acquaintance with musical instruments, and later transitioned into manipulating and performing on “real” instruments, all during the first year. In the second year of the program, they joined a string ensemble for 1 h per week and took part in 3 weekly hours of sectional rehearsals, or, rehearsals arranged by orchestral sections (e.g., first or second violin). In the third year of study, Orff education was phased out. Although our study concerns only the first year of children’s study in the program, understanding some of the tenets of the curriculum provides some insight into the overall music developmental learning goals of the program.

### Procedures

Music related tasks were part of a comprehensive battery of the longitudinal study on the effects of music training on brain, cognitive and social development (Habibi et al., 2014). The focus of the current report is the development of musical skills between induction and the end of the first year. Testing sessions took place at the YOLA at HOLA site or at USC’s Brain and Creativity Institute. All children were tested individually at the start of their participation in the longitudinal study, which, for the music group, coincided with the beginning of their participation in the YOLA at HOLA program. They were tested again approximately 1 year after the initial assessment. Testing took place over multiple short sessions (i.e., typically over 2 or 3 days, depending on individual participants). Children were free to take ample breaks in between individual tasks.

All study protocols were approved by the University of Southern California Institutional Review Board. Parents or legal guardians signed consent forms in their language of choice (i.e., English, Spanish, or Korean) on behalf of child participants. Verbal assent was also obtained from each child at the onset of the study. Parents/legal guardians and children were free to end their participation at any time. Bilingual researchers (Spanish/English, Korean/English) assisted parents and families from the recruitment phase to the scheduling procedures and data collection (see Habibi et al., 2015). Participants (parents/guardians) received monetary compensation for their child’s participation and children were awarded small prizes (e.g., toys and stickers), as a token of our appreciation for their time and effort.

### Assessment of Musical Skills

As noted earlier, the present study examined possible changes in children’s pitch and rhythmic discrimination, pitch matching, singing a song from memory, and rhythmic synchronization skills over the period of 1 year. All tasks were recorded in at least two ways—paper, audio or video—for subsequent data analysis and reliability checks. Testing materials were as follows:

#### Pitch and Rhythmic Discrimination: Primary Measures of Music Audiation

Gordon’s Primary Measures of Music Audiation or PMMA (Gordon, 1986), which is one of the most commonly used standardized tests of tonal and rhythmic perception, was used to assess pitch and rhythmic discrimination at the baseline and in year 1, as there was a small number of participants who were not quite 6 years old at the time of recruitment. Gordon’s PMMA was designed for use by students in kindergarten through third grade. It includes two subtests called *tonal* and r*hythmic*, and requires children to listen to a recording of 40 pairs of simple rhythms (rhythmic test) and 40 pairs of tone sequences (tonal test), and make a same/different judgment for each pair by circling a pair of same or different faces on an answer sheet (Gordon, 1986). Each subtest was administered separately with a total administration time of 20 min. Answer sheets were collected once both subtests were completed.

#### Singing – Pitch Matching: Component 6 from the AIRS Test Battery of Singing Skills (ATBSS)

**FIGURE 1:**
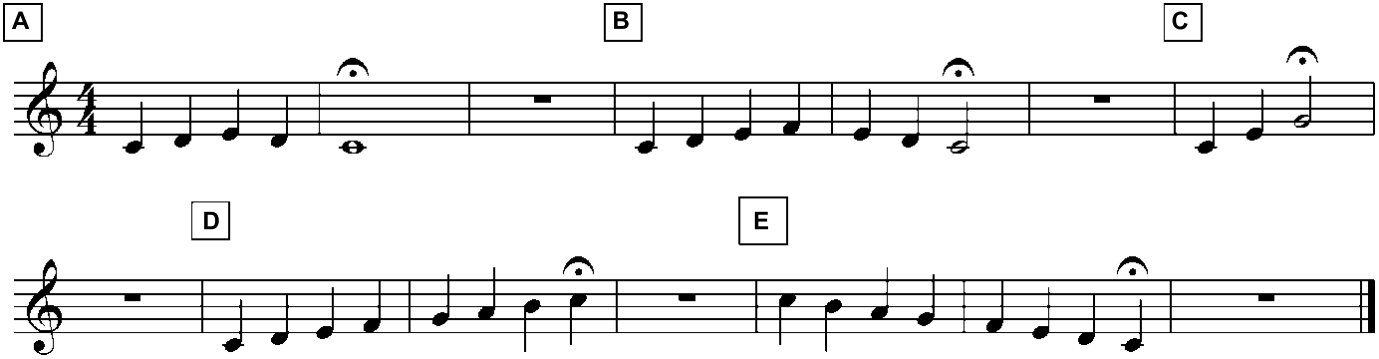
**Score for Component 6 “Musical Elements” from the AIRS Test Battery of Singing Skills**.

Component 6, “musical elements,” from the AIRS Test Battery of Singing Skills or ATBSS^3^ (Cohen et al., 2009) was used to assess children’s pitch matching abilities. Component 6 consists of five musical excerpts involving two melodic patterns in the intervals of a 3rd (C-D-E-D-C), 4th (C-D-E-F-E-D-C), major triad (C-E-G), and scale up (C-D-E-F-G-A-B-C), and down (C-B-A-G-F-E-D-C), using a neutral syllable. A score of all musical excerpts is shown in **Figure 1**. Children were asked to match the pitches of a human model (i.e., a trained singer and researcher) following the presentation of each excerpt. Sung renditions were recorded using a Sony ICD-PX333 digital voice recorder and later extracted for analysis. The software Audacity version 2.0.0 was installed in a Macintosh Computer and used for recording purposes as a backup to the digital recorder.

##### Singing a familiar song from memory

Because different levels of singing competency and different cognitive demands are required in pitch matching and song performance (Mang, 2006), we asked participating children to also sing a familiar song from memory. The selected song was “Happy Birthday,” a well-known song that has been used in previous studies (e.g., Mang, 2006; Pfordresher et al., 2010; Welch et al., 2011; Demorest and Pfordresher, 2015). As in the previous task, children’s sung renditions of “Happy Birthday” were also recorded by the Sony ICD-PX333 digital voice recorded and by Audacity 2.0.0.

#### Rhythmic Synchronization: Drumming

As noted before, rhythmic synchronization is a major milestone of musical development, given its centrality in collective music making, a tenet of collective music programs like El Sistema. For this reason, we measured children’s rhythmic synchronization abilities through drumming, alone and with an adult. Based on previous work by Kirschner and Tomasello (2009) and Kirschner and Ilari (2014), we constructed two identical small padded drums, one for the child participant and one for the experimenter. Inside the participant’s drum, a piezoelectric microphone recorded the response beats. Two different conditions, acoustic and social, were conducted with each participant in a counterbalanced order. For the acoustic condition, prerecorded drumbeats were played via a laptop using Audacity version 2.0.0, a digital audio software program, and amplified through a 12″ guitar amplifier and the participant was asked to drum in synchrony with the pre-recorded beat. In the social condition, overall similar to the acoustic condition, the experimenter played the pre-recorded isochronous beat with the flat palm of his/her hand on the drum and the participant was asked to drum with the experimenter instead of the pre-recorded track. To minimize the interference that the child’s drumming might have on the experimenter’s isochrony, all experimenters were screened for drumming skills and went through multiple training sessions. All sessions were filmed with a digital video (DV) camera to assess reliability of the experiment at a later date.

During drumming sessions, which lasted approximately 5 min in total, the child and the experimenter sat across from each other on two ends of a small table. To familiarize the child with the task of single-handed drumming, during the first session, the experimenter demonstrated a sequence of three beats in the appropriate inter-stimulus interval (ISI) of that session while saying, “Can you also drum this way?” All participants answered this question by imitating the experimenter’s behavior and hitting their drum a few times with only one hand. Then the experimenter sequentially introduced the different tasks by saying the following:

(1) In the acoustic condition: “Look, this is a computer. Listen. There are drum sounds coming out of the computer. Can you drum along with the computer?”
(2) In the social condition: “I want to drum along with you. Listen to how I drum. Can you drum with me?” The order of presentation of the two conditions – acoustic and social – was randomized among participants.

The stimulus files for both the acoustic and social conditions were comprised of evenly spaced stimulus-beats, each with an ISI of 500 ms (120 beats per minute or bpm). In the acoustic condition, a metronome-like click sound (obtained from Audacity) was used as the stimulus-beat; the stimulus file was 57 beats (28 s long). In the social condition, a conga-like drum sample was used as the stimulus-beat, and the stimulus file was 64 beats (32 s long). At the beginning of each stimulus file was an eight beat count-off, which was later excised from both the stimulus and response audio files prior to statistical analysis.

#### Additional Measures

In addition to the abovementioned measures, cognitive abilities data were obtained from testing vocabulary and matrix reasoning (FSIQ-2), using the Wechsler Abbreviated Scale of Intelligence (WASI-II), and motor skills data using the Bruininks-Oseretsky Test of Motor Proficiency (BOT). Because the development of musical skills is known to be contextual (Stadler-Elmer, 2011; Welch, 2016), parents were interviewed on (1) family demographic information, (2) family musical background, (3) use of language in the home, (4) leisure patterns, (5) children’s experiences in formal schooling, and (6) children’s musical habits in everyday life. In this report, we focus on parental/family SES and children’s overall musical habits. [A full analysis of these data is presented elsewhere due to space constraints.]

In the first section of the interview (demographics), parents were asked to indicate their highest level of education and annual household income on a structured questionnaire. Responses to education level were scored on a 5-point scale: (1) Elementary/Middle school; (2) High school; (3) College education; (4) Master’s degree (MA, MS, MBA); (5) Professional degree (Ph.D., MD, JD). Responses to annual household income were also scored on a 5-point scale: (0) < $ 10,000 (1) $10,000 – $19,999 (2) $20,000 – 29,999 (3) $30,000 – 39,999 (4) $40,000 – 49,999 (5) > $50,000. A final socio-economic status (SES) score was calculated as the mean of each parent’s education and annual income scores.

To determine the degree of children’s motivation for and engagement with music in daily life, at the onset of the study, a measure of musical habits was created. This measure was modeled after Kirschner and Ilari (2014) and Ilari and Habibi (2015) and calculated in terms of mean ratings for 10 questions on children’s appreciation of music in daily life and spontaneous and demanded musical behaviors such as singing, listening, and moving (e.g., “My child sings spontaneously”/“My child sings when asked”; “My child enjoys listening to music,” etc.).

## Data Analysis

Data were scored individually for each child on each test and then aggregated by group (i.e., music and control). In the case of standardized tests, namely, WASI-II and BOT, data were analyzed according to the standard norms. WASI-II scores (FSIQ2) were calculated from the two subsections of vocabulary and matrix reasoning of the battery. Data analyses for the specific musical tests were done as follows:

### Pitch and Rhythmic Discrimination: Primary Measures of Music Audiation

The answer sheets for PMMA were scored by recording the number of correct responses in each subtest—tonal and rhythmic—separately. Participants were required to complete 40 items in each subtest. Correct responses received 1 point, with the highest possible score being 40 for each subtest.

### Singing Tasks: Pitch Matching and Singing a Familiar Song from Memory

Singing data were extracted from the digital voice recorder and subsequently analyzed using Rutkowski’s (1998) Singing Voice Development Measure (SVDM). This is one of the most well known rating scales of children’s vocal range (i.e., use of the singing voice). SVDM makes use of a 9-point-scale to describe how children’s singing develops from the (1) “pre singer” to the (5) “singer” stage, with 7 sub-stages in between (Rutkowski, 1998). SVDM is a robust measure of the singing voice across development with reported inter-rater reliability rates of.80 and beyond across many studies (Levinowitz et al., 1998; Rutkowski, 1998; Rutkowski and Miller, 2003). In addition, SVDM can be used to assess children’s singing of sound patterns and intact songs (Ilari and Habibi, 2015), which is consistent with the pitch-matching tasks found in component 6 from the ATBSS.

Individual items in the pitch-matching task were first scored individually and then averaged in a composite score. Two experienced music educators, who were also expert singers, rated all sung renditions for both pitch matching items and “Happy Birthday.” Correlation coefficients were calculated to examine inter-rater reliability for each task and yielded the following results: pitch matching (Pearson *r* = 0.83, *p* < 0.001), and “Happy Birthday” (Pearson *r* = 0.76, *p* < 0.01).

### Rhythmic Synchronization: Drumming

To calculate participants’ instantaneous synchronization accuracy we applied circular statistics (see Kirschner and Tomasello, 2009), to a window of nine consecutive responses beats and moved this analysis window beat by beat across the whole trial. As described in Kirschner and Tomasello (2009), circular statistics allows one to calculate and compare the mean and variance of asynchronies of a sequence of response beats, regardless of their phase direction, that is, whether one child was drumming to the stimulus beat and another child rather off the beat. For each time window, we calculated the mean vector (for calculation see Fisher, 1993; Zar, 1999; Mardia and Jupp, 2000), which can be broken down into two non-parametric components; the vector’s mean direction Θ (‘theta’), which can be used as a measure of the participant’s phase preferences, and the vector’s mean resultant length *R.* The latter varies between zero and one and, in terms of the current analysis, is a direct measure of synchronization accuracy during a particular window, notably independent of the mean direction of beats in that window: an *R* of one would mean perfect synchrony and an *R* close to zero would mean that the child did not actively synchronize his or her movements to the stimulus beat.

For each participant, we averaged the *R* values of each condition. The resulting mean *R* ranges on a linear scale from zero to one. Considering *R* as a measure of how accurate participants were when synchronizing their drumming, we predicted that it should be generally higher in the social conditions compared to the acoustic condition, and group wise higher in the group of children with 1 year if active musical experience (see Kirschner and Tomasello, 2009, 2010). All statistical analyses were performed using the statistical package Statistica for Windows.

To determine whether music perception tasks, as measured by Gordon’s PMMA, correlated with any of the music production tasks (i.e., singing and rhythmic entrainment), two-tailed bivariate Pearson correlation were performed and corrected for multiple comparisons for all the participants collapsed across the two groups.

## Results

### Results at Baseline

The analysis of available data at the time of induction revealed that most families were living with an average family income was of $15,542 (in 78% of the families, and brought in by a single breadwinner), with most parents holding a high school diploma. There were no differences in sex [*c^2^*(1, *N* = 50) = 0.02, *p* = 0.87] between children participants nor any difference in SES [*F*(2,48) = 0.01, *p* = 0.9] between the two groups, and therefore these factors were not included in any subsequent analyses. Age of the children at the onset of the assessment, however, was significantly different between the two groups, *F*(1,48) = 10.23, *p* = 0.002, where children in the control group (mean = 84 months, *SD* = 1.09) were on average 6 months older than the children in the music group (mean = 78.8 months, *SD* = 1.18). No significant correlations were found in two-tailed bivariate Pearson correlations that were performed between age, and performance on all the musical assessments at the onset of the study; therefore subsequent analyses did not include age as a covariate. We first compared the two groups on their performance on each task at baseline (prior to training for the music group) with a series of univariate analyses of variance (ANOVA) with group as independent factor. Results revealed no significant differences between groups on the PMMA tonal or rhythm, pitch matching, sung renditions of “Happy Birthday” or the acoustic condition of rhythm synchronization. In relationship to the US norms, children in the music group scored at the 63rd percentile for the PMMA tonal subtest and at the 72nd percentile for the rhythmic one; whereas children in the control group scored at the 63rd percentile for the tonal subtest and at the 60th percentile for the PMMA rhythmic subtest. In the social condition of rhythm synchronization, however, the music group performed significantly better at baseline than the control group, *F*(1, 30) = 5.6, *p* = 0.02. Participants in the music group also showed higher musical habits as reported by their parents at the onset of the study *F*(1,47) = 11.87, *p* = 0.02. Finally, there was no significant difference between the two groups on test scores for cognitive abilities as measured by Wechsler Abbreviated Scale of Intelligence (FSIQ-2), and motor skills as measured by Bruininks-Oseretsky Test of Motor Proficiency. Equal performance on the two latter assessments provided an equal baseline for the investigation of the development of musical skills in children, irrespective of cognitive or motor abilities. Mean group scores for all measures are presented in **Table 1**.

**Table 1:** Descriptive statistics for all measures: means and standard deviation.

Measure	Music		Control	
	Baseline	Year one	Baseline	Year one
PMMA tonal	30.7 (1.08)	35.8 (0.83)	30.9 (0.95)	33.6 (0.77)
PMMA Rhythm	29.8 (0.98)	30.5 (0.97)	27.5 (0.87)	30.8 (0.91)
Singing – Pitch matching	3.06 (0.27)	3.6 (0.30)	2.7 (0.25)	2.9 (0.27)
Singing – “Happy Birthday”	3.1 (0.18)	3.6 (0.28)	3.1 (0.16)	2.7 (0.25)
Rhythmic synchronization – acoustic	0.84 (0.02)	0.85 (0.03)	0.83 (0.02)	0.76 (0.03)
Rhythmic synchronization – social	0.89 (0.01)	0.90 (0.01)	0.82 (0.02)	0.81 (0.02)
WASI – FSIQ-2 (cognitive abilities)	95.8 (2.62)	94.5 (2.94)	92.03 (2.41)	92.9 (2.71)
BOT (motor abilities)	53.6 (1.93)	54.6 (1.21)	51.6 (1.93)	51.0 (1.11)

### Results at 1 year

Next, we analyzed the data obtained at the end of 1 year after induction. We used a series of repeated measure analysis of variance (ANOVAs) separately with pitch and rhythmic perception (i.e., PMMA), singing from memory and pitch matching (i.e., singing tasks) and rhythmic synchronization (as measured in acoustic and social conditions of entrainment) as separate dependent measures, Group (Music and Control) as between-subject factors, and Year of Assessment (Baseline versus Year one) as within-subject factors. Descriptive statistics for each measure are presented in **Table 2**.

**Table 2:** Group comparisons after one year of music training.

Measure	Results
PMMA – tonal	*F*(1,46) = 0.01, *p* = 0.9
PMMA – rhythm	*F* (1,46) = 3.01, *p* = 0.09
Singing – Pitch matching	*F* (1,41) = 0.59, *p* = 0.44
Singing – “Happy Birthday”	*F*(1,40) = 0.003, *p* = 0.95
Rhythmic synchronization (acoustic condition)	*F*(1,29) = 0.07, *p* = 0.78
Rhythmic synchronization (social condition)	*F*(1,30) = 5.6, *p* = 0.02
WASI – FSIQ-2 (cognitive abilities)	*F*(1,48) = 1.15, *p* = 0.28
BOT (motor abilities)	*F*(1,48) = 061, *p* = 0.43

Concerning PMMA, both groups showed significant increases in rhythmic perception from baseline to year one as evidenced by a main effect of year, *F*(1,45) = 5.35, *p* = 0.02. No significant interaction was observed between task and group was for the rhythm perception task. On the other hand, there were differences between the music group and the control group from baseline to year one in the pitch perception task, main effect of year, *F*(1,46) = 30.42, *p* < 0.001 and a trend toward significance in the interaction of Group × Year *F*(1,46) = 2.98, *p* = 09. *Post hoc* analysis indicated that relative to the control group the music group showed a larger improvement from baseline to year one of in pitch perception (PMMA tonal) moving from 30.7 to 36, whereas the control group went from 30.9 to 33.6. This finding is supported by a strong trend toward significant results of a one way ANOVA on the performance on pitch perception task after 1 year of training with group as independent factor *F*(1,48) = 3.57, *p* = 0.064, where the music group outperformed the control group. Still in regards to PMMA, we compared the scores of child participants from our study with U.S. norms. While children in the music group scored at the 78th percentile for the PMMA tonal subtest and at the 61st percentile for the PMMA rhythm subtest after 1 year of music training, children in control group scored at the 63rd percentile for the PMMA tonal subtest and at the 64th percentile for the rhythm subtest.

In terms of singing skills, both groups improved in the pitch matching task (component 6 from the ATBSS) from baseline to year one, main effect of year, *F*(1,38) = 5.5, *p* = 0.02. There was, however, no significant interaction of Group by Year, although the music group, on average, showed a greater improvement than the control group (3.05–3.65 for music group whereas the control group went from 2.7 to 2.9). These results are displayed in **Table 1**. In relation to sung renditions of “Happy Birthday,” the music group showed a significant improvement from baseline to year one compared to the control group, and there was a significant interaction of Group × Year *F*(1,37) = 8.72, *p* = 0.005. *Post hoc* analyses suggested that compared to the control group, the music group improved from baseline to year 1 of testing whereas the control group had a slight decline in their performance (see **Table 1**).

In regards to rhythmic synchronization, no significant interaction of Group × Year was observed, neither in the acoustic nor in the social condition. In the acoustic condition, however, compared to the control group, the music group improved slightly from baseline to year one of testing whereas the control group declined in their performance. There was no significant difference between the two groups from baseline to year one. A one way ANOVA comparing group performance in the acoustic condition of the rhythmic synchronization task after 1 year of training showed a strong trend toward significance *F*(1,30) = 3.54, *p* = 0.06 with the music group outperforming the control group. As for the social condition of the rhythmic synchronization task, the music group performed significantly better than the control group *F*(1,30) = 10.94, *p* = 0.002 after 1 year of training – of note given the difference between the two groups at the onset, the difference at 1 year was larger than the difference at the onset.

Significant correlations, after Bonferroni correction, were found between performance on PMMA tonal and pitch matching (*r* = 0.31; *p* = 0.012) and between PMMA-tonal and sung renditions of “Happy Birthday” (*r* = 0.37; *p* = 0.002) after 1 year of music training. Regarding correlations between PMMA (tonal and rhythmic) and entrainment, the only significant correlation, after Bonferroni correction, was between performance on PMMA tonal and rhythmic synchronization in the social condition at the baseline assessment (*r* = 0.4; *p* = 0.007). No other significant correlations were found.

## Discussion

Results from our study suggest that participation in an El Sistema-inspired program, over the course of 1 year, had an impact on children’s musical development, particularly in the development of pitch schemata. Children in the music group outperformed control children in the PMMA tonal test, which involves frequency discrimination, pitch change detection and pitch direction (Fancourt et al., 2013). These findings are consistent with previous studies that found associations between music learning and pitch processing (e.g., Orsmond and Miller, 1999; Schellenberg and Moreno, 2010; Roden et al., 2014). Children in the music group were also more proficient than their control peers in a task that involved producing pitches in a sequence to form a familiar melody (i.e., “Happy Birthday”). It is also interesting that, although they did not reach statistical significance, improvements were seen in the music group in terms of pitch matching tasks (component 6 from the ATBSS). This finding is consistent with the notion that different pitch perceptual abilities develop at different rates. As noted earlier, while both frequency discrimination and pitch change detection reach adult levels when children are about 6–7 years of age, sensitivity to pitch direction takes longer to develop, becoming adult-like only when children reach 10 or 11 years of age (Trainor and Corrigall, 2010; Fancourt et al., 2013). So, while all participating children were developing pitch perceptual abilities as they are expected during typical development, music training might be regarded as contributing to accelerated pitch discrimination skills in those children who were attending the music program.

But why did children in the music group show more improvements when singing an intact familiar song (i.e., “Happy Birthday”) than for a pitch matching test? One plausible explanation may be related to the specific characteristics of the singing tasks. While “Happy Birthday” was rated only once and according to SVDM norms, there were five scores for the pitch matching tasks that were later averaged to yield the final score. Additionally, two items in the pitch matching task were known to be difficult for developing singers, namely, singing a scale up and singing a scale down (see Gooding and Standley, 2011). Anecdotal evidence suggested this to be true for some children, who struggled to reach high pitches or appeared to be “lost” in the pitch sequence in one or both directions, as voiced by our experimenters. We have videoed all testing sessions and are now analyzing them qualitatively, to get a better understanding of children’s performance on these specific tasks. Therefore it is possible that lower ratings for these difficult items were the reason why pitch matching scores were lower than those attributed to renditions for “Happy Birthday.” A second possible explanation for these disparate singing results may be related to the nature of instrumental group learning. Children who are learning how to play musical instruments are often encouraged to sing melodies to themselves as a way to internalize the music that will be performed. Singing back short melodies to a teacher/conductor (as it occurs in the pitch matching tasks), on the other hand, is usually not common in the practice of instrumental learning, particularly in an orchestral setting. Therefore, it seems possible that the novelty associated with the pitch matching tasks may have affected the responses of children in the music group. In any case, it will be interesting to see how children, who continue in the music program, will perform in the pitch matching tasks over time. Importantly, it should be noted that, for both groups, children’s average SVDM scores, which are indicative of children’s use of the singing voice or vocal range (Rutkowski, 1998, 2015), were around 3, the *Limited range singer* level in the SVDM classification. This is consistent with previously reported results (Levinowitz et al., 1998; Rutkowski and Miller, 2003; Ilari and Habibi, 2015).

Perhaps, the fact that children in the music group outperformed control children in their sung renditions of “Happy Birthday” could be explained as a simple effect of near transfer (Singley and Anderson, 1989). Roden et al. (2014, p. 554) have suggested that transfer in music is more prone to occur in connection to “cognitive functions strongly related to auditory functions,” like verbal memory and phonological loop abilities. Consistent with previous research (see Welch et al., 2011), music training has been associated with improvements in verbal memory (Chan et al., 1998; Forgeard et al., 2008). Likewise, singing a familiar song also makes demands on long-term memory, including verbal memory (see Pfordresher et al., 2015). Thus it is possible that through ensemble activity children also fine-tuned their musical memory for familiar tunes. This assertion needs to be substantiated by future research.

As in other studies that were based on Gordon’s audiation tests (e.g., Forgeard et al., 2008; Roden et al., 2014), children’s group scores for the rhythmic test were lower than for the tonal one, and developmental effects were stronger than training effects. In other words, all children improved their rhythmic discrimination skills over the course of a year, and so did the children in the music group, performing at equivalent levels to controls in year 1. These results also allow for some speculation. First, it is possible that the development of pitch schemata precedes the development of rhythmic schemata. Second, there may be a discrepancy between the rhythmic and the tonal subtests, with the former being slightly more difficult than the latter for both PMMA and IMMA. Future studies could examine whether this is true by testing musically trained and untrained children in cultures whose music shows more variance in terms of rhythmic and metric structures. Alternatively, it is also possible that the focus of children’s music education program played a role in the results. As noted, study participants were introduced to the violin during their first year in the intensive El Sistema-inspired program. Given the melodic nature of this instrument and the fact that string players must generate pitches (as there are no frets in the instrument), teachers often emphasize pitch perception in the early years of learning. This, in turn, may have affected our results. Future longitudinal studies could compare the development of rhythmic and tonal discrimination skills in different modalities of music learning (e.g., string players, pianists, singers) during middle childhood.

Based on prior research (Drake et al., 2000; Slater et al., 2013), we expected children in the music group to show larger improvements in the rhythmic synchronization task than their control peers. Yet, this was clearly not the case. It is important to recall that children in the music group started off with stronger rhythmic synchronization skills than controls in the social task. Although children in the music group were admitted by means of a lottery, they self selected to be in this collective music education program by the choice of their parents to apply in the first place. It is possible that children who signed up for the El Sistema-inspired music program were either predisposed or simply more motivated to play/engage with others in a social setting; this fact may have resulted in the better rhythmic synchronization scores. This explanation is corroborated by parental ratings of children’s motivation and interest in music, gathered from the parental interviews. Children in the music group also showed higher ratings in terms of musical interests at the baseline than their control peers. Interestingly, while rhythmic synchronization scores for children in the music group remained constant or slightly improved after 1 year of training, those of the control group declined, particularly in the acoustic condition. These last findings suggest that musical development was taking place. Musical development, after all, does not necessarily follow a linear path but rather combines periods of rapid growth with periods of learning plateaus and “disequilibrium and sensitivity” to a growing complexity and interaction between, symbol systems, hearing abilities and musical experiences (see Bamberger, 2005, p. 73). Therefore, the fact that children in the music group maintained equivalent scores after 1 year and could sustain their attention and synchronize to the beat is remarkable; particularly if one considers the task, which has to be described essentially as a “boring” task in the acoustic condition, involving tapping an isochronous beat sequence with a computer, for a long time and, furthermore, is no longer a novel task.

Overall, it was interesting to see that, while the musical skills of children in the music group either stayed constant or improved over the course of 1 year of training, the musical skills of children in the control group tended to decline after 1 year. With the exception of rhythmic perception and pitch matching, other measured skills such as singing a song from memory and synchronizing to the musical beat decreased for controls. Beyond the commonsensical idea that music training enhances the development of musical skills (e.g., Fujioka et al., 2006; Trainor and Corrigall, 2010), our findings seem to suggest that a lack of musical training may actually promote the decline of some specific musical skills that are developed throughout the child’s life, through enculturation alone or in combination with formal training. This interpretation will, of course, need to be formally evaluated in the future. One way to do this is through the examination of more than one music education program, which was not done in the current study. Moreover, we cannot rule out the possibility that our findings are simply indicative of participants’ motivation (or lack thereof) to complete the designated musical tasks.

It is also important to consider our findings in light of children’s socioeconomic status and parental support, which are two related issues that are known to influence early music learning and development (McPherson, 2009; Lareau, 2011; Ilari and Young, 2016). In comparison to more aﬄuent families, low-income families are said to provide less intellectual stimulation, even when parental education, family structure, race and ethnicity are controlled (Doob, 2015), and this is partly due to the many economic and psychological stresses that they face. Additionally, engagement in formal music education such as school orchestras, choirs and bands in the United States, has been more commonly associated with middle and upper class students than students from lower SES (see Elpus and Abril, 2011). Child participants in our study were, for the most part, living below poverty levels (the threshold being at approximately U$24,000 for a family of four, as suggested by the Public Policy Institute of California, 2015), in underserved areas of Los Angeles. Unsurprisingly, many parents reported serious financial, work-related, family (i.e., single parenthood), and housing challenges during the interview. Based on these assumptions, some would probably expect children from our study to perform at lower levels than what has been documented in research with their more aﬄuent peers. Our results, however, suggest otherwise. Overall, the development of pitch and rhythmic skills of our study participants undergoing music education aligned with findings from previous studies conducted with more aﬄuent children (Forgeard et al., 2008), as well as same aged children from Germany (Roden et al., 2014). PMMA scores for both tonal and rhythmic tests were consistent with the U.S. norm. Children’s uses of the singing voice (Rutkowski, 1998), as measured through SVDM, were also comparable to findings from prior studies conducted with middle class children in North America, South America and Western Europe. While these results could be due to the intensity of the studied music program—arguably more intense than average,—they could also be explained by other factors. For example, interview data (not reported here due to space limitations) suggested that parental support of children’s music learning was high. This aligns with the idea that parents who enroll their children in music programs may be more engaged with their education (see Forgeard et al., 2008), which may result in gains in musical and non-musical domains. Children in our study also showed high levels of enthusiasm about learning music formally during their first year in the program, as seen in non-participant observations of classes and rehearsals, and in our conversations with them. Parental interview data further suggested that children from both groups had similar music listening preferences and habits (e.g., singing and dancing to pop tunes), yet very few had ever attended a live musical performance. It will be interesting to probe these issues in the forthcoming years with families who continue in the study, to better understand how they relate to the development of musical skills.

Another issue to interrogate is the selection of measurement tools when researching the development of musical skills in childhood. As Welch (2009, p. 149) contended, “research endeavors are likely to be better placed to effect change if we locate them in real world situations (which is not as easy as it might seem), or ensure that they have an appropriate applicability to such situations.” The non-significant results for the pitch matching tasks in the music group after 1 year of training, not supporting prior findings (e.g., Welch et al., 2014b), could be explained in terms of a mismatch between the task at hand and everyday activities in the music program. Due to the fact that little research exists concerning the musical development of children from non-WEIRD populations and that no specific test results have been validated for such populations, we purposefully selected measures that have been used earlier, e.g., Gordon’s audiation tests (e.g., Roden et al., 2014) and the ATBSS (e.g., Ilari and Habibi, 2015). If, on the one hand, these measures afforded us with opportunities to contrast our findings with available results, on the other, it is possible that they may have lacked ecological validity (Welch, 2009).

Along the same lines, our study reinforces the need to thoroughly define music training in future research, as learning and development are intertwined (Bamberger, 2005). Music training is sometimes treated in a “monolithic” way, creating a false logic that participation in any program will necessarily develop children’s “general” musical skills in a predetermined or uniform fashion (for a discussion see Rauscher and Hinton, 2011). Yet programs differ considerably from one another, even when they share common philosophical and methodological underpinnings (e.g., Osborne et al., 2015). Program characteristics including the intensity of music training, aligned with children’s overall development and predispositions toward music, SES, cultural background and family support are likely to influence children’s musical development, as we believe happened in our study. On that note, we are currently analyzing qualitative data that were collected alongside with the quantitative data described in this report. Data obtained through interviews with parents, unstructured interviews with teachers and children, field observations, children’s instrumental performances and vocal improvisations, will be presented in separate reports due to space limitations.

In summary, our results are consistent with the idea that, far from being linear, musical development is a complex, multifaceted, spiraled and recurring process (Bamberger, 2005) that is based on a changing dynamics of “growth, maintenance, and loss” (Gembris, 2006, p.128). Periods of rapid intellectual growth are often interspersed with periods of learning plateaus in musical development (Zimmerman, 1986). Our findings also align with the idea that musical skills develop over time, with some possibly taking longer to develop, depending on the quality and intensity of musical training coupled with maturation of other related areas. Results from our study indicate that the perception of pitch, which emerges early in ontogeny, is enhanced by just 1 year of intensive music training during middle childhood. That is, there could be a hierarchy in the development of musical skills, with pitch skills developing earlier than rhythmic skills, particularly in children who learn Western “art” music, like in the El Sistema-inspired program that we have studied. Most importantly, our findings suggest that different musical skills develop in childhood based on the combination of children’s individual interests, parental support, formal music training, and everyday musical experiences, with social markers like socioeconomic status playing less important roles. Not only is this consistent with the wide range of childhood musical practices found across the globe (e.g., Campbell and Wiggins, 2013), but it also provides additional evidence in support of the provision of quality music education programs for all children.

## Author Contributions

BI: main author: design of the work, data collection, analysis, interpretation and report write up; PK: data collection, analysis, interpretation. HD: design of the work, interpretation, report write up. AH: design of the work, analysis, interpretation and write up.

## Conflict of Interest Statement

The authors declare that the research was conducted in the absence of any commercial or financial relationships that could be construed as a potential conflict of interest.

## Footnotes

1 The Youth Orchestra of Los Angeles at Heart of Los Angeles program is a partnership between the Los Angeles Philharmonic Association and the organization Heart of Los Angeles. For additional information, please refer to http://www.laphil.com/education/yola/hola

2 The term Latino is used particularly in the West Coast of the United Stated, in reference to migrants from Latin America who have Spanish as their native language (Valencia, 2004). Rather than being a nationality, the term Latino is applied to over 20 nationalities, and is related to different ethnic and cultural groups.

3 THE ATBSS the AIRS Test Battery of Singing Skills or ATBSS (Cohen et al., 2009; Cohen, 2015) is a comprehensive instrument consisting of 11 components that include: (1) opening conversation; (2) determine vocal range; (3) minor third participant-name-call-back; (4) Sing back “Brother John” song all, as eight phrases, and all; (5) sing favorite song; (6) sing back interval, triad, scale, herein called, *musical elements*; (7) improvise ending of a song; (8) free composition to choice of picture; (9) Sing back unfamiliar song “We are one” by Carolyn McDaid, (10) sing “Brother John” from recent memory; and (11) closing conversation. Each component serves one or more specific research purposes as indicated by Cohen et al. (2009). It was designed to acquire data from varied age, cultural and ethnic backgrounds. The ATBSS can be administered by a trained researcher using recorded prompts or through its online version.
